# Decreased RNA polymerase III subunit expression leads to defects in oligodendrocyte development

**DOI:** 10.3389/fnins.2023.1167047

**Published:** 2023-04-25

**Authors:** Julia Macintosh, Mackenzie Michell-Robinson, Xiaoru Chen, Geneviève Bernard

**Affiliations:** ^1^Department of Neurology and Neurosurgery, McGill University, Montreal, QC, Canada; ^2^Child Health and Human Development Program, Research Institute of the McGill University Health Center, Montreal, QC, Canada; ^3^Department of Pediatrics, McGill University, Montreal, QC, Canada; ^4^Department of Human Genetics, McGill University, Montreal, QC, Canada; ^5^Department of Specialized Medicine, Division of Medical Genetics, McGill University Health Center, Montreal, QC, Canada

**Keywords:** RNA polymerase III, leukodystrophy, oligodendrogenesis, glia, hypomyelination

## Abstract

**Introduction:**

RNA polymerase III (Pol III) is a critical enzymatic complex tasked with the transcription of ubiquitous non-coding RNAs including *5S* rRNA and all tRNA genes. Despite the constitutive nature of this enzyme, hypomorphic biallelic pathogenic variants in genes encoding subunits of Pol III lead to tissue-specific features and cause a hypomyelinating leukodystrophy, characterized by a severe and permanent deficit in myelin. The pathophysiological mechanisms in POLR3- related leukodystrophy and specifically, how reduced Pol III function impacts oligodendrocyte development to account for the devastating hypomyelination seen in the disease, remain poorly understood.

**Methods:**

In this study, we characterize how reducing endogenous transcript levels of leukodystrophy-associated Pol III subunits affects oligodendrocyte maturation at the level of their migration, proliferation, differentiation, and myelination.

**Results:**

Our results show that decreasing Pol III expression altered the proliferation rate of oligodendrocyte precursor cells but had no impact on migration. Additionally, reducing Pol III activity impaired the differentiation of these precursor cells into mature oligodendrocytes, evident at both the level of OL-lineage marker expression and on morphological assessment, with Pol III knockdown cells displaying a drastically more immature branching complexity. Myelination was hindered in the Pol III knockdown cells, as determined in both organotypic shiverer slice cultures and co-cultures with nanofibers. Analysis of Pol III transcriptional activity revealed a decrease in the expression of distinct tRNAs, which was significant in the siPolr3a condition.

**Discussion:**

In turn, our findings provide insight into the role of Pol III in oligodendrocyte development and shed light on the pathophysiological mechanisms of hypomyelination in POLR3-related leukodystrophy.

## Introduction

Hypomyelinating leukodystrophies encompass a group of genetic white matter disorders in which there is a failure of central nervous system (CNS) myelination in development (Vanderver et al., 2015; Wolf et al., 2021). They present in previously healthy children and lead to progressive neurodegeneration and premature death (Vanderver et al., 2015). While hypomyelinating disorders were historically thought to be due to defects in genes that encode structural myelin proteins, the canonical example being Pelizaeus-Merzbacher disease resulting from pathogenic variants in *PLP1*, the genetic etiology of these heterogenous disorders has since broadened (Wolf et al., 2021). Indeed, more recent advances have identified a subset of hypomyelinating leukodystrophies that arise from defects of transcription and translation machinery, such as RNA polymerase III-related hypomyelinating leukodystrophy (POLR3-related leukodystrophy). Now known to be one of the most common hypomyelinating leukodystrophies, POLR3-related leukodystrophy arises from biallelic pathogenic variants in genes that encode subunits of RNA polymerase III (Pol III), namely *POLR3A*, *POLR3B*, *POLR1C,* and *POLR3K* (Bernard et al., 2011; Tétreault et al., 2011; Thiffault et al., 2015; Dorboz et al., 2018; Schmidt et al., 2020).

Pol III is a 17-subunit enzymatic complex responsible for the transcription of ubiquitous and essential RNAs, including *5S* rRNA, all tRNA genes, and a variety of functionally distinct non-coding RNAs (ncRNAs) such as *7SL*, which serves as a scaffold for the signal recognition particle and *U6*, a component of the spliceosome (Dieci et al., 2007). Pathogenic variants in genes associated with POLR3-related leukodystrophy are diverse and include missense, nonsense, small insertions or deletions, exonic or intronic splice site variants, and large exonic deletions (Bernard et al., 2011; Tétreault et al., 2011; Daoud et al., 2013; Wolf et al., 2014; Gutierrez et al., 2015; Thiffault et al., 2015; La Piana et al., 2016; Bernard and Vanderver, 2017; Gauquelin et al., 2019). Notably, there are no reports of living individuals harbouring two null alleles of any of the affected genes, suggesting that some residual Pol III function is requisite for life. The mapping of pathogenic variants to the human Pol III structure has revealed clustering of variants at subunit interfaces, predicted to affect the biogenesis and/or stability of the polymerase (Ramsay et al., 2020; Girbig et al., 2021). Pathogenic variants have also been shown to impair nuclear import of the complex or impede its ability to bind DNA for transcription, while functional work has shown reduced protein expression of the affected Pol III subunit in fibroblasts and brain lysates in affected individuals (Bernard et al., 2011; Thiffault et al., 2015).

Despite advances in the genetic etiology of this disorder, the pathophysiological mechanisms remain enigmatic. POLR3-related leukodystrophy is known to have a spectrum of disease severity, ranging from a very severe form that presents in the first few months of life and leads to rapid decline and premature death, to a mild form diagnosed incidentally on magnetic resonance imaging (MRI) (Wolf et al., 2014; DeGasperis et al., 2020; Perrier et al., 2020). POLR3-related leukodystrophy is likewise recognized as 4H leukodystrophy for its characteristic features of hypomyelination, hypodontia, and hypogonadotropic hypogonadism. Neurological features, stemming from hypomyelination, often present as motor delays or regression and can include cerebellar (e.g., ataxia, dysmetria, dysarthria, cerebellar tremor), pyramidal (e.g., spasticity) and extrapyramidal (e.g., dystonia) features (Osterman et al., 2012; Wolf et al., 2014; Al Yazidi et al., 2019; Gauquelin et al., 2019). Variable cognitive involvement is also seen and may involve learning difficulties, intellectual disabilities, and/or cognitive regression (Wolf et al., 2014; Gauquelin et al., 2019). Meanwhile, non-neurological features include abnormal dentition, myopia, and endocrine abnormalities, such as hypogonadotropic hypogonadism with absent, delayed, or arrested puberty (Wolf et al., 2014; Gauquelin et al., 2019; Pelletier et al., 2021).

Associated with the most debilitating aspect of disease, the hypomyelination seen in patients remains poorly understood. Myelin, produced by oligodendrocytes (OLs) in the CNS, serves a crucial function in protecting neurons and enabling saltatory conduction for efficient propagation of action potentials (Nave and Werner, 2014). Previous work on the histopathology of POLR3-related leukodystrophy supports a primary involvement of OLs in the pathogenesis of the classic form of the disease (Vanderver et al., 2013; Wolf et al., 2014). OLs undergo a complex developmental process to become myelinating cells, beginning with an oligodendrocyte precursor cell (OPC) migrating significant distances to reach its final site of myelination before commencing proliferation (Michalski and Kothary, 2015; van Tilborg et al., 2018). Eventually becoming post-mitotic as it differentiates, an immature OL will then undergo drastic increases in morphological complexity to enable contact with numerous axons for subsequent myelination (Michalski and Kothary, 2015).

Recent work in a mouse model of *Polr3a* identified a reduction in mature, MOG+ OLs, which the authors proposed could be due to a defect in oligodendrogenesis (Merheb et al., 2021). As a detailed study of OL maturation in model organisms is complicated by the spatiotemporal complexity of OL-lineage development *in vivo*, we sought to characterize how reduced Pol III impacts OL development *in vitro*. In this study, we downregulated endogenous transcript levels of leukodystrophy-associated Pol III genes, namely *Polr3a, Polr3b,* and *Polr1c*, using small interfering RNAs (siRNA) in primary mouse OL-lineage cells in order to model the hypomorphic effect of variants in a cell-type predicted to be dysfunctional in disease. We then subjected these cells to developmental processes that recapitulate their development *in vivo* (e.g., migration, proliferation, differentiation, and myelination) to shed light on a role for Pol III in OL maturation and on POLR3-related leukodystrophy pathophysiology.

## Materials and methods

### Animals

C57BL/6 mice were obtained from Charles River Laboratory Canada. All procedures with animals were approved by the Animal Resource Division of the RI-MUHC (protocol #2018–8055) and performed in accordance with the Canadian Council on Animal Care Guidelines for animal use in research.

### Mouse brain primary OPC cultures

OPCs were isolated from P6 wildtype (C57BL/6) mice of either sex using a modified version of immunopanning, as described in Macintosh et al. (Emery and Dugas, 2013; Macintosh et al., 2023). Briefly, mice were cryo-anesthetized and brain tissue, specifically telencephalon and mesencephalon, dissected before dissociation in papain, L-cysteine, and DNase I for 90 min at 37°C with perfusion of 95% O_2_/5% CO_2_ gas. Post-dissociation, the digested brain tissue was pelleted before gently triturating in 10X ovomucoid solution, a papain inhibitor, to form a single-cell suspension. Cells were spun down (220 g, 15 min, RT) and resuspended in 6X ovomucoid, followed by resuspension in immunopanning buffer (D-PBS supplemented with 0.2% BSA and 0.5 mg/ml insulin). The resulting cell suspension was filtered and sequentially passed onto 2x 10cm Petri dishes coated with bandeiraea simplicifolia lectin (15 min, RT) to which microglia and endothelial cells adhere. The residual cell suspension was then passaged onto a positive selection plate coated with an anti-PDGFRα antibody (45 min, RT). The plate was rinsed with D-PBS and the adhered cells were dissociated with trypsin to be spun down. OPC pellets were resuspended in serum-free OPC proliferation media (DMEM with 1X GlutaMAX, 1 mM sodium pyruvate, 5 μg/mL insulin, 5 μg/mL N-Acetyl-L-cysteine, 1X trace elements B, 1X penicillin–streptomycin, 10 ng/mL biotin, 10 µg/mL BSA, 10 µg/mL transferrin, 1.6 µg/mL putrescine, 60 ng/mL progesterone, 40 ng/mL sodium selenite, 4.2 μg/mL forskolin, 1X B-27 (-vitamin A), 10 ng/mL PDGF-AA and 10 ng/mL bFGF) and seeded onto poly-D-lysine (PDL; 50 μg/mL) coated 10 cm plates. OPCs were maintained in a 10% CO_2_ incubator and by supplementing with PDGF-AA (10 ng/mL) at 2 days *in vitro* (DIV) followed by half media changes every 2 days starting at 4 DIV. For experimental objectives involving differentiation, cells were passaged and plated in differentiation media (+T3, 40 ng/mL, -PDGF-AA, -bFGF).

### OPC/OL siRNA transfection

Primary OPCs were passaged at 7 DIV into media (+PFGF-AA/bFGF or + T3/-PDGF-AA/-bFGF, depending on experimental objectives) containing Lipofectamine® RNAiMax transfection reagent (Invitrogen) and 15 pM siRNA (Dharmacon siRNA SMARTPool: *Polr3a, Polr3b,* or *Polr1c*) prepared following manufacturer’s instructions at a siRNA to Lipofectamine volume:volume ratio of 1:1. The level of knockdown was assessed through RT-qPCR using the ∆∆Ct method with three reference genes (*18s, Gusb, Pgk1*). A non-targeting siRNA (siGENOME siRNA pool #2) was used as a negative control in respective experiments.

### Immunofluorescence

Cells (OPCs/OLs) were fixed in 4% paraformaldehyde. Cells labelled for OPC markers were collected after 48 h on coverslips in proliferation media, while cells labelled for OL markers were plated on coverslips in T3/differentiation media for either 48 h (O4; immature OL marker) or 72 h (MOG and MBP; mature OL markers). After washing, cells were blocked in blocking buffer (PBS with 5% BSA, 0.2% Triton X-100) for 1 h at RT. Samples were then washed and incubated with primary antibodies (Supplementary Table S1; rat anti-PDGFRα (BD, 1:100), rabbit anti-NG2 (Millipore, 1:200), sheep anti-Ki67 (Novus, 1:200), mouse anti-O4 (R&D, 1:200), rat anti-MOG (R&D, 1:300), rat anti-MBP (Novus, 1:100), rabbit anti-cleaved caspase 3 (CST, 1:250) in blocking buffer for 2 h at RT. After washes, cells were incubated for 2 h at RT with DAPI and corresponding secondary antibodies (Supplementary Table S1; AlexaFluor 488 donkey anti-rat (Jackson ImmunoResearch, 1:1000), AlexaFluor 647 donkey anti-rabbit (Jackson ImmunoResearch, 1:300–500), AlexaFluor 594 donkey anti-sheep (Abcam, 1:500), AlexaFluor 555 donkey anti-goat (Invitrogen, 1:500), AlexaFluor 594 donkey anti-mouse (Jackson ImmunoResearch, 1:500). For O4, labelling was done live at 37°C with 10% CO_2_, cells were blocked for 1 h with 5% FBS-DMEM and then incubated with primary antibody in 5% FBS-DMEM for 2 h. For labelling of slice cultures, slices were fixed in 4% PFA (1 h, on ice). Following washes, slices were treated with 5% glacial acetic acid (20 min) and washed. Slices were blocked in blocking buffer (3% horse serum, 2% BSA, 0.25% TX-100, and 0.1% sodium azide; 2 h, RT) before incubation with primary antibodies (rat anti-MBP (Novus, 1:100) and anti-Calbindin D28k (Swant, 1:5000) in primary incubation buffer (2% BSA, 0.25% TX-100, 0.1% sodium azide) for 20 h at RT. Slices were washed and incubated with respective secondary antibodies (overnight, RT). Following washes, slices were mounted with Immu-Mount. All immunolabelling was imaged using an EVOS Epifluorescent microscope or Zeiss LSM780 laser scanning confocal microscope and processed using ImageJ or ZEN microscope software.

### RNA extraction and reverse transcription quantitative polymerase chain reaction

Cells (OPCs/OLs) were lysed in TRIzol reagent (ThermoFisher) and total RNA extracted using a miRNeasy kit (QIAGEN), following manufacturer’s instructions and with on-column DNase I treatment (Qiagen). For the first few extractions, RNA was run on a bleach gel to confirm quality and effectiveness of the DNase I treatment in removing genomic DNA. RNA concentration and quality were assessed on a ND-1000 Nanodrop spectrophotometer. For each sample, 500 ng of RNA was reverse transcribed with M-MLV reverse transcriptase (Promega) and a 1:1 mix of Oligo(d)T (ThermoFisher) and random hexamer primers (IDT). Primers were designed to span exon-exon junctions using the Primer-Blast NCBI and OligoAnalyzer IDT software (Supplementary Table S2). All primers were validated using cDNA derived from mouse brain tissue to meet MIQE guidelines (Bustin et al., 2009; Taylor et al., 2019). cDNA was amplified using a Roche LightCycler® 96 instrument using the following parameters: pre-incubation (95°C for 180 s), 3-step amplification (95°C for 10s, 60°C for 30s, 72°C for 30s; 45 cycles) and melting (95°C for 10s, 65°C for 60s, 97°C for 1 s) and expression assessed using SsoAdvanced Universal SYBR green supermix (Bio-Rad Laboratories). Gene expression of each biological replicate was analyzed in technical triplicates using the ∆∆Ct method with a 1:40 cDNA dilution and normalized to three reference genes (*18s*, *Gusb,* and *Pgk1*). Expression was presented as relative to the negative control condition.

### Transwell insert migration assay

OPCs were resuspended in base media (OPC media -PDGF-AA/-bFGF) at a density of 1.25 × 10^5^ cells/mL and 100 μL was seeded into the upper chamber of a poly-D-lysine coated 8 μm pore polycarbonate transwell insert (Corning™ Transwell™ Multiple Well Plate with Permeable Polycarbonate Membrane Inserts, Fisher) similarly to previously described protocols (Frost et al., 2000; Jarjour and Kennedy, 2004). The insert was placed over a well of a 24-well tissue culture plate containing 600 μL of base media and 20 ng/mL PDGF-AA. OPCs were left to migrate through pores in the insert for 16 h at 37°C. A cotton swab was used to remove non-migrating OPCs remaining in the upper chamber, before fixing cells that had migrated to the lower chamber in 4% PFA (30 min, RT). Following rinses with PBS, nuclei were stained with DAPI. For each transwell insert, 3–6 images were taken of a random 4X objective field on an epifluorescent microscope. For quantitative assessment, cells were counted using the analyze particles function on ImageJ software, with cell counts from each insert averaged. Three independent experiments were performed for all conditions.

### OPC EdU uptake experiment

As a means to assess OPC proliferation, transfected OPCs were treated with Click-IT™ EdU Cell Proliferation Kit (ThermoFisher) for imaging as per manufacturer’s protocol. Briefly, OPCs were seeded onto 15 mm coverslips and transfected in proliferation media. Forty-eight hours post-transfection, OPCs were treated with 10 μM EdU solution and incubated for another 20 h. Following the EdU incubation, cells were fixed (4% PFA in PBS, 10 min, RT), washed (3% BSA in PBS, 2× 10 min, RT), permeabilized (0.5% TX-100 in PBS, 20 min, RT) and incubated with Click-IT™ reaction cocktail to detect EdU labelling (30 min, RT). After washing, cells were stained with DAPI and mounted with Immu-Mount (ThermoFisher). Each coverslip was imaged in 5 randomly chosen 20X objective fields using an epifluorescent microscope. EdU and DAPI signal were counted using the analyze particles function on ImageJ software and the amount of EdU+ cells (as a percentage of DAPI) were averaged for each coverslip. Five independent experiments were performed for all conditions.

### Morphological analysis

As a measure of morphological complexity, OPCs transfected with siRNA (control or Pol III targeting) and passaged into T3-containing differentiation media were fixed at 2 DIV (O4) or 3 DIV (MOG) post-transfection and labelled. Immunolabelling was imaged using a Zeiss LSM780 laser scanning confocal microscope. The category of OL maturity was determined based on (1) OL with minimally branched processes, (2) OL with complex branched processes, and (3) OLs that form partial membrane-like sheaths, as previously described (Barateiro and Fernandes, 2014). Oligodendrocyte processes were traced using the Simple Neurite Tracer Plugin on Fiji (ImageJ) and the render/analyze skeletonized paths function used for the schematized reconstruction. For quantitative analysis, field area (i.e., area within the contours of the processes created by connecting the outermost tips of distal processes) and total branch length (i.e., sum of the length of all branches per OL) were measured. Process branching complexity was further quantified using the concentric ring analysis Plugin on ImageJ for Sholl analysis (Gensel et al., 2010). As an overview, a series of concentric rings were superimposed on the cell, starting at the soma (parameters: inner radius 20uM, outer radius 140uM, step size 10 μM), and the number of intersections at each ring was determined. For MOG analysis, four independent experiments for each condition were used, in which five to eight cells were analyzed per coverslip. For O4 analysis, three independent experiments for each condition were used, in which three cells were analyzed per coverslip.

### ReadyProbe live/dead assay

To look at cell death, cells were treated with a cell-permeable NucBlue® live reagent (Hoescht 33342) and cell-impermeable NucGreen® dead reagent using the ReadyProbe assay kit (ReadyProbes™ Cell Viability Imaging Kit, Blue/Green). Fluorescence signal was imaged using a DAPI and GFP filter on the EVOS epifluorescent microscope and cell counts quantified using the analyze particles function on ImageJ. Percentage of cell death was determined by the number of dead (GFP+) cells divided by the sum of dead and live (DAPI+) cells. Data is presented as a fold-change of the control condition.

### Genotyping Shiverer mice

Tails were taken from mice prior to dissecting cerebellums and DNA extracted using the Extracta DNA prep for PCR (QuantaBio) according to manufacturer’s instructions. Shiverer mice were genotyped with a multiplex PCR using previously described primers: WT band flanking exon 6 (deleted in *Shi*/*Shi* mice) [5′ - AGCTCTGGTCTTTCTTGCAG – 3′ and 5′ – CCCCGTGGTAGGAATATTACATAAC – 3′] and primers complementary to the shiverer breakpoint [5′ – CAGGGGATGGGGAGTCAGAAGTGAG – 3′ and 5′ – ATGTATGTGTGTGTGTGCTTATCTAGTGTA – 3′] (Gomez et al., 1990). PCR was performed with Taq polymerase (Promega) and the cycling conditions were as follows: 3 min at 94°C, 35 cycles of 45 s at 94°C, 45 s at 60°C, 1 min at 95°C and 10 min at 72°C. For genotype analysis, PCR products were separated using agarose gel electrophoresis (1.5% agarose gel; 120 V for 30 min) and imaged using a Doc EZ System (BioRad).

### Shiverer organotypic slice cultures

Cerebellar slices from shiverer mice were cultured and transplanted with OPCs, as previously described (Bin et al., 2012). Briefly, shiverer mice at P0 were cryo-anesthetized and cerebellums dissected and sectioned into 300 μm sagittal sections with a McIlwain tissue chopper. Slices were transferred onto Millicell inserts (MilliporeSigma™ Millicell™ Culture Plate Inserts, Fisher) in serum-containing media (MEM with Earle’s salts, EBSS, heat-inactivated horse serum, 1X GlutaMAX, 1X penicillin–streptomycin, 6.25 mg/mL glucose, 0.5 μg/mL Fungizone). Media changes were performed every 2 days and media was progressively changed from serum-containing media to serum-free media (DMEM/F-12, 1X GlutaMAX, 1X B-27, 1X N2, 1X penicillin–streptomycin, 0.5 μg/mL Fungizone). At 21 DIV, purified siRNA-treated OPCs (Ctrl siRNA, siPolr3a, siPolr3b, siPolr1c) were injected into the slice (20000 OPCs/slice in 2× 0.5 μL injections). Slices were maintained in serum-free media for a further 3–6 weeks, at which time slices were fixed (4% PFA, 1 h on ice) and labelled using methods described under immunofluorescence. Analysis of MBP+ area as a percentage of total Calbindin area was performed on the 10X objective field images and determined using the threshold and the analyze particle functions on Fiji (ImageJ). Data from three independent experiments for each condition were used, in which a minimum of 2 replicate slices were analyzed.

### Nanofiber myelination assay

MimitexA® 96-well plates for 3D cell culture from AMSBIO were first activated in 70% ethanol before washes and treatment with PDL coating (50 μg/mL, 2 h at 37°C). Nanofibers were then rinsed 3x with water (5 min) and 1x with DMEM neat. Cells transfected with siRNA (control or Pol III target) were collected and resuspended for seeding at 2.4×10^4^ in 200 μL per well. Cells in 3D-culture were maintained with media changes, in T3/differentiation media, every 2 days and collected at 14 DIV by fixing in 4% PFA for 15 min. Cells were immunolabelled in the plate, starting with blocking (5% BSA, 0.2% TX-100) at RT for 1 h and with primary antibody (rat anti-MBP, Novus, 1:100) overnight at 4°C. Following washes, the 3D cultures were incubated with secondary antibody (AlexaFluor 488 donkey anti-rat, Jackson ImmunoResearch, 1:1000) for 2 h at RT before washing and imaging in plate (Zeiss LSM780 laser scanning confocal microscope). Length analysis was quantified using the line measure tool on Fiji (ImageJ).

### Graphs and statistical analysis

Data analysis was performed using GraphPad Prism 9.1.1. Quantifications were performed from at least three independent experiments. Statistical analysis was performed using a Student’s *t*-test to compare between two groups. Statistical significance was set at *p < 0.05. Data is shown as mean ± SEM. Two-toned heatmaps were made using the heatmap function on GraphPad Prism, using the normalized fold expression value.

A detailed list of reagents used in this study can be found in Supplementary Table S3.

## Results

### Decreasing Pol III subunit expression in OPCs increases the proportion of actively dividing cells

**Figure 1:**
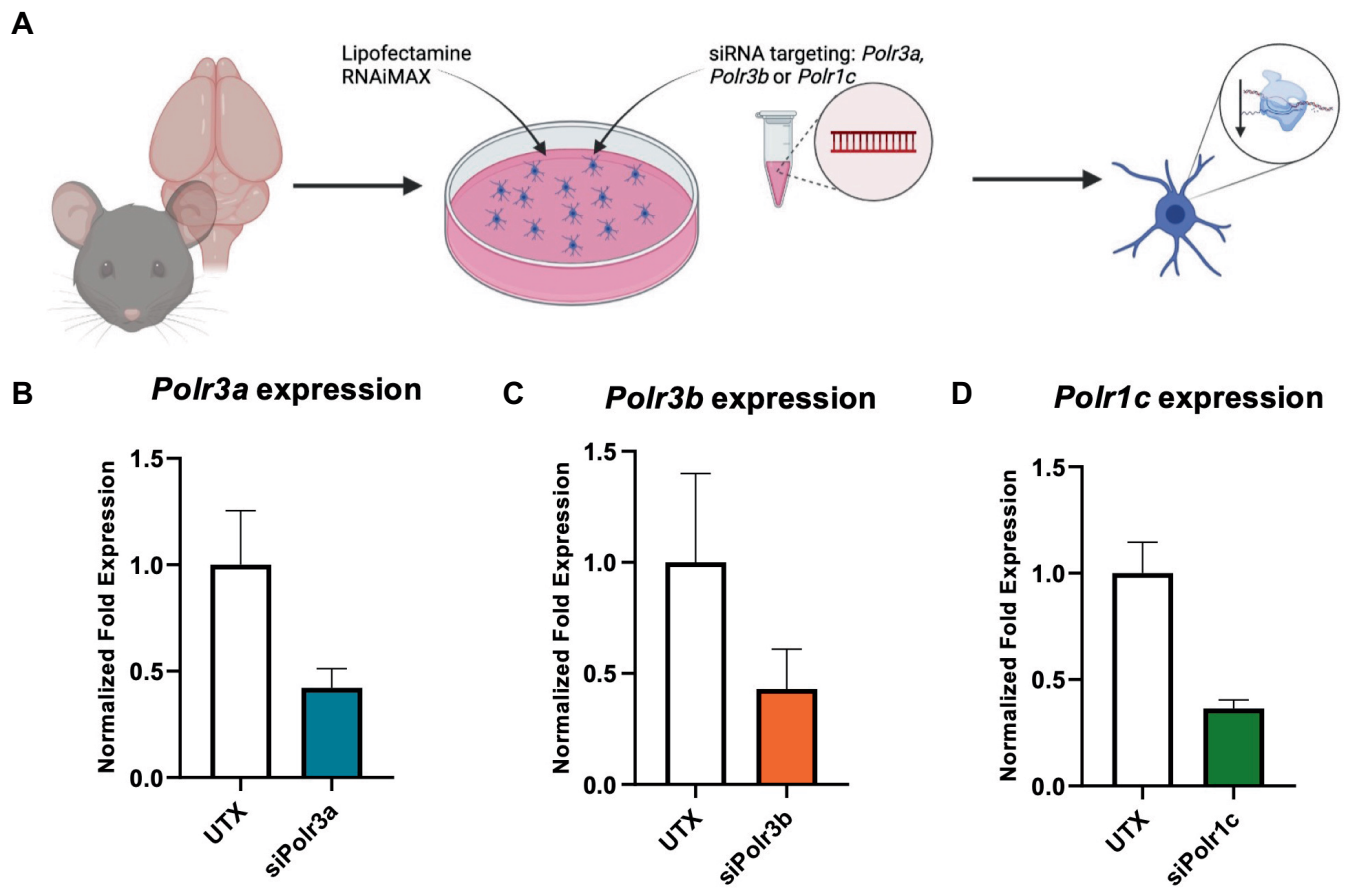
Characterization of Pol III expression following siRNA treatment. **(A)** Schematic of mouse OPC isolation and siRNA treatment. **(B–D)** RT-qPCR analysis of OPCs subjected to either **(B)** siPolr3a, **(C)** siPolr3b, or **(D)** siPolr1c knockdown conditions with resulting expression of the respective Pol III transcript graphed (*Polr3a* 58% reduced, *Polr3b* 57% reduced, *Polr1c* 63% reduced). Data represents mean ± SEM normalized fold expression after normalizing to reference genes *18 s, Gusb,* and *Pgk1.*

**Figure 2:**
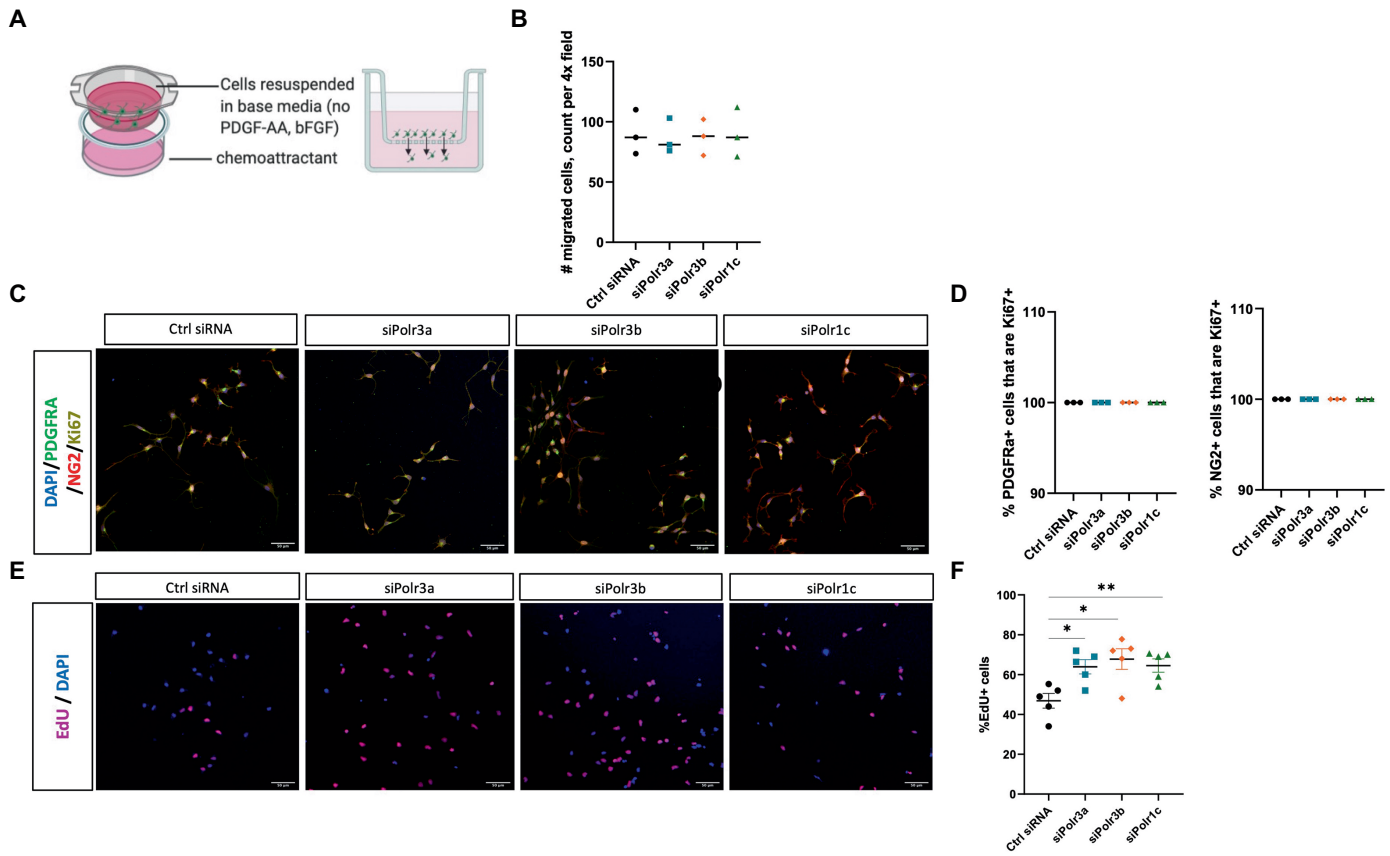
Decreased expression of leukodystrophy-associated Pol III subunits in OPCs increases the proportion of proliferating cells but does not affect migration. **(A)** Schematic of the transwell insert migration assay. **(B)** Quantification of siRNA-treated OPC migration, as determined by cell count in a randomly imaged 4X field. Data represents mean ± SEM from three independent experiments (each presented as an average of 3–6 technical replicates). **(C)** Representative immunofluorescence images (from three independent experiments with similar results) of siRNA-treated OPCs in proliferation media, showing PDGFRα (green), NG2 (red), and Ki67 (yellow). Nuclei stained with DAPI. Scale bar, 50 μm (20X). **(D)** Quantification of the % PDGFRα+ (left) and NG2+ (right) cells that are Ki67+ from three independent experiments. **(E)** Representative images (from five independent experiments with similar results) of siRNA-treated OPCs labelled with EdU. Nuclei stained with DAPI. Scale bar, 50 μm (20X). **(F)** Quantification of EdU+ cells as a percentage of DAPI following siRNA-treatment. Data represents mean ± SEM from five independent experiments (each presented as an average of 5 technical replicates), *p < 0.05, **p < 0.01, unpaired, two-sided Student’s *t*-test.

To study the role of Pol III in a precursor stage of OL development, we transfected OPCs with siRNA targeting *Polr3a, Polr3b* or *Polr1c*. We were able to achieve a consistent downregulation between Pol III conditions, decreasing endogenous expression of Pol III genes by an average of 60% (range 57–63%; Figure 1). Migration of OPCs during development is required for the cells to reach their axon targets, a failure in which could result in a myelin deficit (Michalski and Kothary, 2015). As such, we assessed whether downregulation of disease-associated Pol III subunits impacts migration of OPCs by subjecting the cells to a transwell migration assay (Figure 2A). We found the number of migrated cells under PDGF-AA chemoattractant-driven conditions comparable between the treatment and control conditions, suggesting migration was unaffected in the context of Pol III downregulation (Figure 2B).

We next assessed the role of Pol III in the proliferation of OPCs by immunolabelling for the proliferation marker Ki67 (Scholzen and Gerdes, 2000). Triple immunolabelling of OPCs in proliferation media for Ki67, PDGFRα, and NG2 showed that Pol III siRNA-treated and control cells were likewise positive for Ki67 (Figure 2C; Supplementary Figure S1). When we quantified the proportion of PDGFRα+ or NG2+ cells (OPC markers) that were Ki67+, there was no difference between the treatment and control conditions, with all PDGFRα+ and NG2+ cells positive for Ki67 (Figure 2D). As Ki67 is a non-specific marker of proliferation, we next performed an EdU uptake experiment to look directly at cells actively undergoing DNA synthesis and to further assess the possibility of altered OPC proliferation in the context of decreased Pol III expression. In line with the Ki67 immunolabelling, all EdU+ cells were likewise positive for PDGFRα and/or NG2 (Supplementary Figure S2). To our surprise, we found there were more EdU+ cells in the Pol III siRNA-treatment conditions than in the control (Figure 2E; Supplementary Figure S3). Indeed, when we quantified the proportion of EdU+ cells, we found the ratio of OPCs undergoing DNA synthesis was significantly higher in the Pol III siRNA-treated conditions relative to the control, signifying an actively proliferating cell population (Figure 2F). Overall, these findings indicated that decreasing Pol III expression during the precursor stage of the OL-lineage has no effect on migration but may augment the rate of OPC proliferation.

### Pol III is required for differentiation of OPCs into OLs

**Figure 3:**
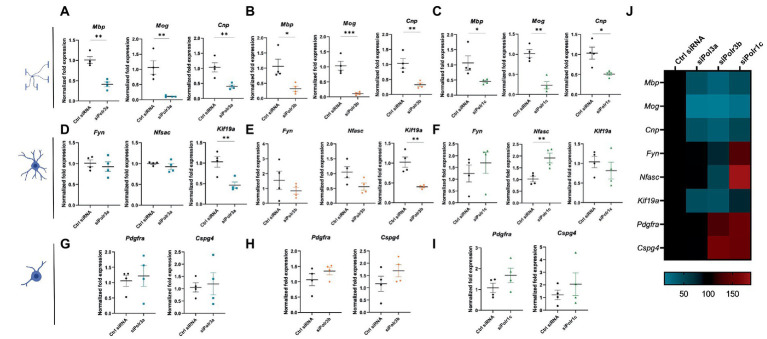
Reducing expression of Pol III subunits impairs expression of mature OL genes. **(A–C)** RT-qPCR analysis of RNA derived from siRNA-treated OLs passaged into T3-containing differentiation media and collected at 3 DIV, demonstrating expression of myelin genes *Mbp, Mog,* and *Cnp* in **(A)** siPolr3a, **(B)** siPolr3b and **(C)** siPolr1c conditions. **(D–F)** RT-qPCR analysis of RNA derived from siRNA-treated OLs passaged into T3-containing differentiation media and collected at 3DIV, demonstrating expression of immature OL genes *Fyn, Nfasc,* and *Kif19a* in **(D)** siPolr3a, **(E)** siPolr3b, and **(F)** siPolr1c conditions. **(G–I)** RT-qPCR analysis of RNA derived from siRNA-treated OLs passaged into T3-containing differentiation media and collected at 3DIV, demonstrating expression of OPC genes *Pdgfr*α and *Cspg4* in panel **(G)** siPolr3a, **(H)** siPolr3b, and **(I)** siPolr1c conditions. Data represents mean ± SEM normalized fold expression after normalizing to reference genes *18 s, Gusb,* and *Pgk1.* *p < 0.05, **p < 0.01, ***p < 0.001, unpaired, two-sided Student’s *t*-test. **(J)** Heatmap representation of normalized fold expression, determined by RT-qPCR, grouped by OL-lineage marker (row), assessed across all siRNA-treatment conditions (column).

To characterize the role of Pol III in OL differentiation, we performed RT-qPCR analysis on RNA collected from cells after 3 days in differentiation media. Analysis of myelin marker gene expression demonstrated Pol III subunit transcript downregulation was associated with significantly reduced expression of *Mbp, Mog,* and *Cnp*, findings that were consistent across all Pol III conditions (Figures 3A–C). We reasoned that the reduction in myelin transcripts could be due to a decrease in the production of myelin or a defect in differentiation, in which case the cells would be too immature to produce transcripts enriched in late stage OLs. To assess for blocked maturation, we next looked at the expression of developmental marker transcripts enriched in immature OLs including *Fyn*, *Nfasc,* and *Kif19a*, involved in cytoskeletal activity, organizing the nodes of Ranvier, and microtubule binding activity, respectively (Figures 3D–F; Richter-Landsberg, 2008; Pillai et al., 2009; Zhang et al., 2014; Wang et al., 2016). We found *Kif19a* to be significantly decreased in the siPolr3a and siPolr3b conditions. In comparison, *Nfasc* and *Fyn* expression were more variable. While non-significantly decreased in the siPolr3a and siPolr3b conditions, both transcripts were increased in the siPolr1c condition, with *Nfasc* significantly increased. In comparison, there was a slight increase in expression of early OPC markers *Pdgfr*α and *Cspg4* (i.e., gene that encodes the OL marker NG2) in all Pol III siRNA-treated cells under differentiation conditions, though non-significant (Figures 3G–I). Altogether, while our results at the immature OL stage were more variable between conditions, our findings at the OPC and mature OL stage were consistent between Pol III siRNA conditions (Figure 3J), emphasizing the critical contribution of each subunit to the function of the entire enzymatic complex. Overall, these results suggest that decreasing Pol III subunit transcript expression in OL-lineage cells is likely to impair OL maturation.

### Reduced Pol III expression stunts OL maturation without causing cell death

**Figure 4:**
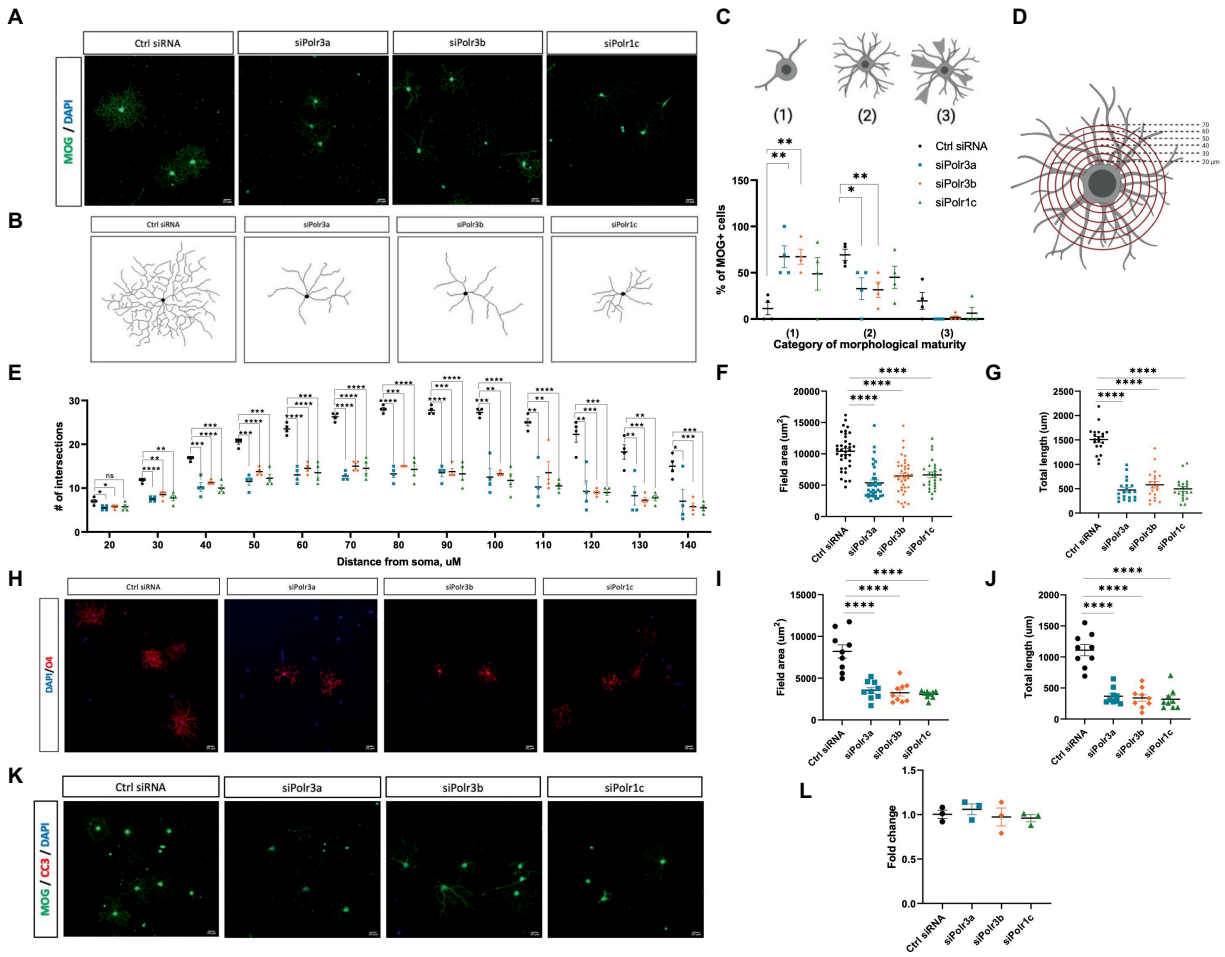
Reduced morphological complexity of cells with decreased expression of Pol III subunits. **(A)** Representative immunofluorescence images (from four independent experiments with similar results) of siRNA-treated OLs in differentiation conditions, showing MOG+ cells (green). Nuclei stained with DAPI. Scale bar, 20 μm (20X). **(B)** Representative examples of OL processes from control or Pol III siRNA-treated cells as schematized diagrams. **(C)** Categorization of OL morphological maturity, as determined by branching complexity and myelin-like sheath formation, of MOG+ cells collected after 3DIV in differentiation media. **(D)** Schematic of Sholl analysis, in which a series of concentric rings are super-imposed on the cell as a measure of process networks (parameters - inner radius 20 μM, outer radius 140 μM, step size 10uM). **(E)** Quantification of Sholl analysis, depicted as mean ± SEM number of intersections at each ring from four independent experiments (each presented as an average of 5–8 technical replicates). *p < 0.05, **p < 0.01, ***p < 0.001, ****p < 0.0001, unpaired, two-sided Student’s *t*-test. **(F,G)** Quantification of **(F)** field area and **(G)** total length analysis in siRNA-treated cells labelled with MOG. **(H)** Representative immunofluorescence images (from three independent experiments with similar results) of siRNA-treated OLs in differentiation conditions, showing O4+ cells (red). Nuclei stained with DAPI. Scale bar, 20 μm (20X). **(I,J)** Quantification of **(I)** field area and **(J)** total length analysis in siRNA-treated cells labelled with O4. **(K)** Representative immunofluorescence images (from three independent experiments with similar results) of siRNA-treated OLs in differentiation conditions, showing MOG+ cells (green) immunolabelled for CC3 (red). Nuclei stained with DAPI. Scale bar, 20 μm (20X). **(L)** Percent cell death as determined by ReadyProbe assay. Data presented as fold change of control condition graphed as mean ± SEM.

Given our findings of reduced expression of late-stage OL transcripts in the Pol III siRNA conditions, we performed immunocytochemistry analysis to evaluate the morphological complexity of these cells and further assess the possibility of impaired maturation. Immunofluorescence labelling for MOG under differentiation conditions revealed that Pol III siRNA-treated cells retained a more immature branching network than their control-transfected counterparts, exemplified by the corresponding branching diagrams (Figures 4A,B). Categorizing cells into stages of maturation based on morphology revealed that there were more OLs with poorly-branched processes and fewer OLs with branched processes (i.e., complex OLs) in Pol III siRNA-treated cells, which was seen in all conditions and significant for the siPolr3a and siPolr3b conditions (Figure 4C). Few cells producing membrane-like sheaths (category 3) were found in all conditions, including the Ctrl siRNA, likely due to a brief period of exposure to differentiation media (3 DIV) before analysis. To further quantify the difference in morphological complexity, we evaluated process branching using Sholl analysis (Figure 4D), which confirmed that the Pol III siRNA-treated cells had a less elaborate network of processes than the control cells (Figure 4E). This difference could be seen at the level of primary processes (i.e., the processes that protrude directly from the soma) in the siPolr3a and siPolr3b conditions (Figure 4E; 20 μm ring – siPolr3a: *p = 0.024, siPolr3b: *p = 0.04, siPolr1c: *p* = 0.09), but was most substantial at the level of process branching (30+ μm from soma) (Figure 4E). While the control cells demonstrated an intricate branching complexity, the Pol III siRNA-treated cells showed, on average, significantly fewer intersections at each concentric ring (Figure 4E). Additionally, at the most distant concentric ring, superimposed 140 μm from the soma, the control condition had significantly more intersections than the Pol III conditions (Figure 4E; 140 μm ring – siPolr3a: *p = 0.038, siPolr3b: ***p = 0.0009, siPolr1c: ***p = 0.0006). This suggested that in addition to having a more immature branching complexity, the Pol III siRNA-treated OLs had fewer long processes, and/or that the processes were on average shorter. Further quantitative analysis revealed the field area and total length of processes in Pol III siRNA-treated cells were drastically decreased relative to control-treated cells (Figures 4F,G). To ensure our findings were not due to poor expression of MOG, we performed immunocytochemistry analysis by labelling with O4, an immature OL marker, which confirmed these cells had an immature branching complexity (Figures 4H–J).

Oligodendrocyte differentiation is known to be regulated via a bottleneck process, with a substantial proportion of immature OLs lost to apoptosis during development (Hughes and Stockton, 2021). As such, we sought to determine whether impaired differentiation in the Pol III siRNA-treated cells subjected them to apoptosis. Immunofluorescent analysis of Pol III siRNA-treated OLs after 3 days in differentiation conditions showed they did not label for cleaved-caspase 3, a marker of apoptosis (Figure 4K). To further assess the possibility of cell death in these cells, we treated cells with NucBlue™ live reagent (Hoechst 33342) and NucGreen™ dead reagent as part of a ReadyProbe assay to determine the percentage of cell death. While cell death was seen in all conditions, it was comparable in the treatment and control conditions (Figure 4L). Taken together, these findings support the notion that reducing Pol III subunit transcript expression impairs differentiation of OLs, causing them to retain a more immature morphology, but that these cells nonetheless remain alive.

### Decreased levels of Pol III subunits impair myelination

**Figure 5:**
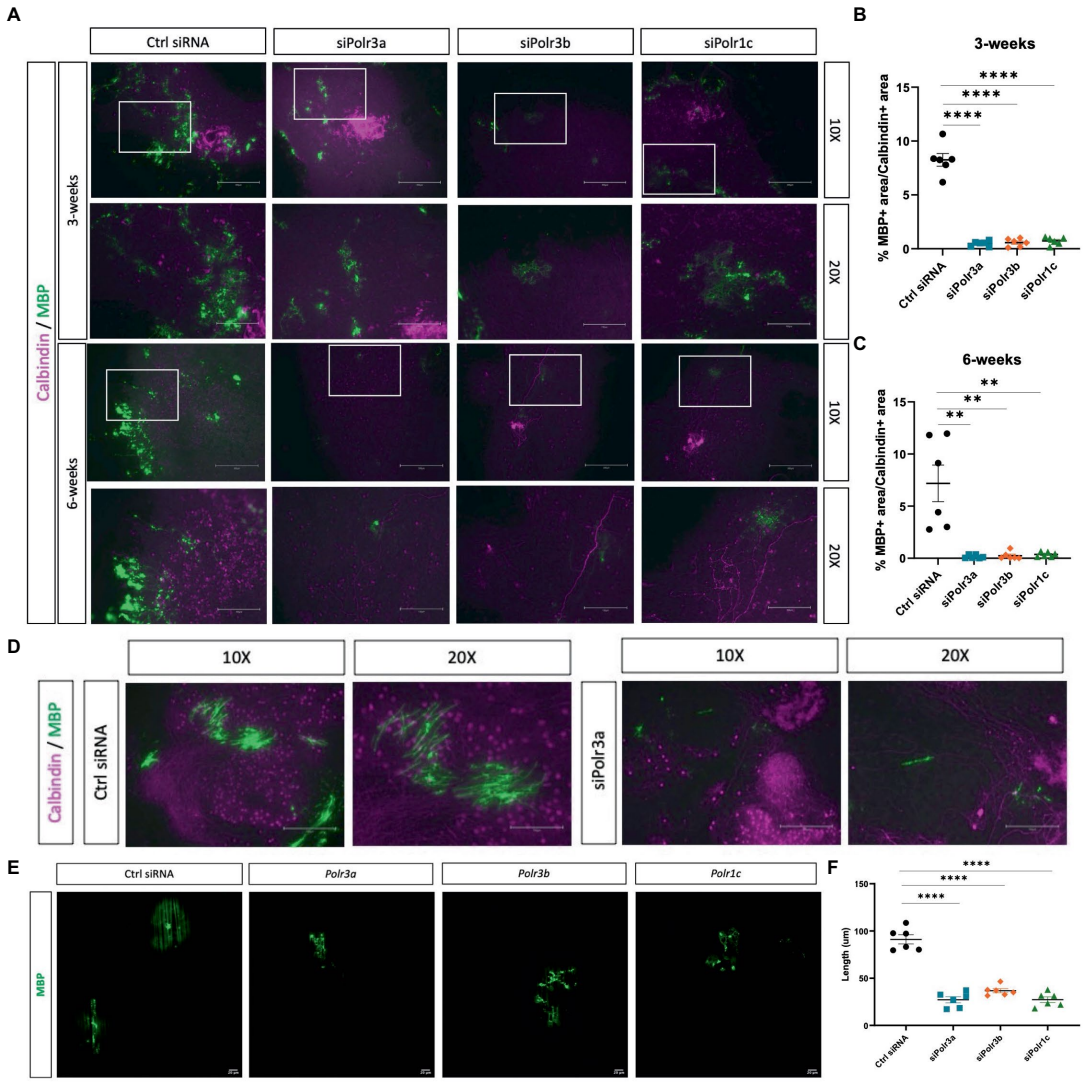
Reducing Pol III expression in OLs impairs myelination. **(A)** Representative immunofluorescence images (from three independent experiments per timepoint with similar results) of *Shi/Shi* organotypic slices transplanted with siRNA-treated OPCs (Ctrl siRNA, siPolr3a, siPolr3b, siPolr1c) showing Calbindin (magenta) and MBP (green) collected at 3 weeks or 6 weeks. Scale bar, 300 μm (10X), 150 μm (20X). **(B,C)** Quantitative analysis of organotypic shiverer slice culture assay, with % MBP+ area/Calbindin+ area depicted as mean ± SEM from three independent experiments (each with 2 technical replicates), determined from 10X objective at **(B)** 3- and **(C)** 6-week timepoints. **p < 0.01, ****p < 0.0001. **(D)** Immunofluorescence images of Shi/Shi organotypic slices transplanted with siRNA-treated OPCs (Ctrl siRNA, siPolr3a) in an axon-rich cross-section. Collected at 6 weeks. Scale bar, 300 μm (10X), 150 μm (20X). **(E)** Representative immunofluorescence images (from three independent experiments with similar results) of siRNA-treated OPCs cultured with nanofibers showing MBP (green). 20X objective, scale bar, 20 μm. **(F)** Quantification of MBP+ sheath length depicted as mean ± SEM from three independent experiments (each with 2 technical replicates). ****p < 0.0001, unpaired, two-sided Student’s *t*-test.

Higher-order branching of OLs is a requirement for the cell to function in myelinating numerous axons (Michalski and Kothary, 2015). Considering the findings of impaired maturation, we next looked at how a decrease in Pol III subunit expression might impact the ability of OLs to myelinate. To assess myelination in an *ex vivo* micro-environment, we generated organotypic slice cultures from the homozygous Shiverer mouse and transplanted siRNA-treated OPCs into the slice cultures, before fixing slices at 3- and 6-week timepoints to analyze their myelination capacity as inferred by MBP signal (Readhead and Muggleton-Harris, 1991). Immunofluorescence labelling for MBP at 3 weeks revealed fewer MBP+ regions in the slices with Pol III siRNA-treated cells, as compared to the condition with control cells (Figure 5A). This finding of fewer MBP+ regions was consistent at the 6-week timepoint (Figure 5A), suggesting the impaired myelination state persisted. Next, we quantified the MBP+ area by performing a % calculation of MBP+ area/Calbindin+ area, which uncovered that the % of MBP+ area was significantly reduced in all conditions relative to the negative control and that this finding was consistent at both the 3- and 6-week timepoints (Figures 5B,C). On occasion, cross-sections of subcortical cerebellar regions, in which Purkinje axons project to the deep nuclei, can be seen in organotypic shiverer slice cultures. We were able to find such axon-rich areas for both the control and the siPolr3a treatment condition at the 6-week timepoint, which revealed a visual insufficiency of MBP+ axon ensheathment in the siPolr3a condition, relative to control (Figure 5D).

As an additional assessment of myelination capabilities, we co-cultured Pol III siRNA-treated cells with nanofibers, biologically inert fibers that act as synthetic axons. We observed that while the control-treated cells were able to extend MBP+ sheaths along the length of the nanofibers, MBP+ could be found in the Pol III treated cells but these sheaths were less organized along the fiber (Figure 5E). In line with this, and similar to what was observed in the organotypic shiverer slice culture assay, when we quantified the length of MBP+ sheaths formed by OLs in each condition, we found the Pol III siRNA-treated cells extended MBP+ sheaths along the fibers that were drastically shorter than the control condition (Figure 5F). Altogether, these 3D analyses extended our understanding of the deficit in OL membrane and process elaboration observed in 2D cultures, and confirmed that Pol III also plays a critical role in myelinogenesis.

### Decreasing Pol III subunit expression in OL-lineage cells alters tRNA transcription, coinciding with a defective oligodendrogenesis state

**Figure 6:**
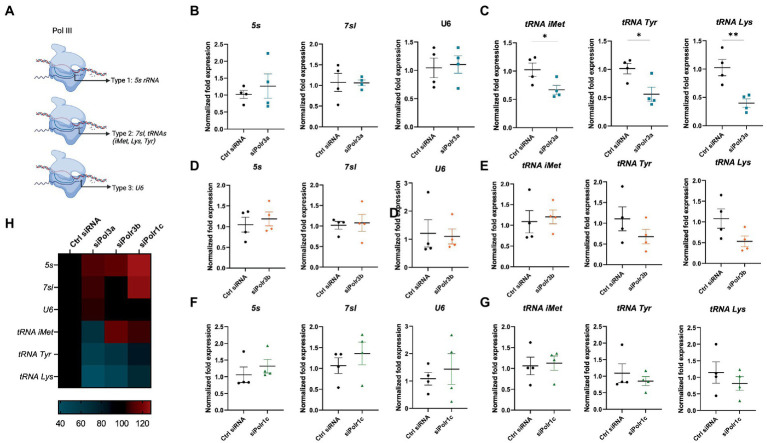
Decreasing Pol III subunit expression in OL-lineage cells alters Pol III transcription. **(A)** Schematic of Pol III transcription, as grouped by its three promoter types. **(B,C)** RT-qPCR analysis of RNA derived from siPolr3a-treated OLs passaged into T3-containing differentiation media and collected at 3DIV, demonstrating expression of Pol III transcripts **(B)** *5 s, 7sl,* and *U6,* and **(C)** tRNAs: tRNA-iMet (CAT 1-1), tRNA-Tyr (GTA 1-1), and tRNA-Lys (TTT 2-1). **(D,E)** RT-qPCR analysis of RNA derived from siPolr3b-treated OLs passaged into T3-containing differentiation media and collected at 3DIV, demonstrating expression of Pol III transcripts **(D)** *5 s, 7sl,* and *U6,* and **(E)** tRNAs iMet (CAT 1-1), Tyr (GTA 1-1), and Lys (TTT 2-1). **(F,G)** RT-qPCR analysis of RNA derived from siPolr1c-treated OLs passaged into T3-containing differentiation media and collected at 3DIV, demonstrating expression of Pol III transcripts **(F)** *5 s, 7sl,* and *U6*, and **(G)** tRNAs; tRNA-iMet (CAT 1-1), tRNA-Tyr (GTA 1-1), and tRNA-Lys (TTT 2-1). Data represents mean ± SEM normalized fold expression after normalizing to reference genes *18 s, Gusb,* and *Pgk1.* *p < 0.05, **p < 0.01, ***p < 0.001, unpaired, two-sided Student’s *t*-test. **(H)** Heatmap representation of normalized fold expression, determined by RT-qPCR, grouped by OL-lineage marker (row), assessed across all siRNA-treatment conditions (column).

To assess what might be occurring at the level of Pol III activity in the cells displaying these differentiation and myelination deficits, we performed RT-qPCR analysis of Pol III transcripts in siRNA-treated cells (Ctrl siRNA, siPolr3a, siPolr3b, siPolr1c) subjected to differentiation conditions. Specifically, as a proxy for each Pol III promoter type (Figure 6A), we assessed the expression levels of *5s, 7sl,* and *U6* RNA following Pol III subunit downregulation. We observed no significant differences in the expression of these transcripts (Figures 6B,D,F). Since tRNAs have previously been implicated in the pathophysiology of POLR3-related leukodystrophy (Azmanov et al., 2016; Dorboz et al., 2018; Choquet et al., 2019), we next assessed the levels of certain distinct tRNAs (Figures 6C,E,G). While the expression of tRNA-iMet-CAT-1-2 varied between conditions, it was significantly decreased in the siPolr3a condition. Further, tRNA-Tyr-GTA-1-1 and tRNA-Lys-TTT-2-1 were decreased in all the Pol III siRNA-treatment conditions and these decreases were significant in the siPolr3a condition (Figures 6C,E,G). Overall, despite similarities in our morphology results, we did observe variation between Pol III siRNA conditions in our transcriptional analysis (Figure 6H). Altogether, these results elucidated Pol III transcriptional defects that coincide with and help explain our cellular findings.

## Discussion

RNA polymerase III-related leukodystrophy is a devastating neurodegenerative condition. Despite the neuropathological evidence from human autopsy samples suggesting that OLs are primarily involved in disease pathogenesis (Vanderver et al., 2013; Wolf et al., 2014), how the complex development of OLs is altered in the disease remains poorly understood. As such, it is unclear whether the hypomyelination seen in patients arises from a singular failure of OPCs to migrate, proliferate, differentiate, or myelinate, or if a combination of mechanisms is at play. Here, we utilized a downregulation approach to reduce expression of leukodystrophy-associated Pol III subunits in primary murine OL-lineage cells and then subjected these cells to comprehensive *in vitro* assays that recapitulate their developmental stages to shed light on the pathogenic mechanism of hypomyelination observed in POLR3-related leukodystrophy.

As the biallelic pathogenic variants seen in affected patients are hypomorphic, we opted to use a siRNA-based knockdown approach to target Pol III subunits known to be affected in POLR3-related leukodystrophy. We achieved a fairly consistent reduction of approximately 60% of Pol III subunit transcript expression. Nevertheless, as this percentage of knockdown is based on bulk extracts, these results likely reflect a combination of the number of cells targeted by the lipofectamine-siRNA complex and the degree of knockdown within those cells. It is unclear, in patients, what level of transcript or protein expression may be associated with an affected Pol III gene. Past work in HEK293 cells engineered to carry the M852V *POLR3A* variant demonstrated a *POLR3A* mRNA decrease of 20–60%, depending on whether the missense variant was in combination with a frameshift mutation or a null allele (Choquet et al., 2019). Analysis of protein expression in patients has suggested similar reductions in patient samples (Bernard et al., 2011; Perrier et al., 2020). Prior mouse studies have proposed that embryonic viability requires >15% of wildtype Pol III activity in mice, while Pol III dysfunction is known to manifest phenotypically in both yeast and mice at approximately 30% of the level of wildtype activity (Wang et al., 2020; Merheb et al., 2021). Taken together, we expect that alteration of Pol III expression below 50% of normal is likely to cause disruptions to cell function, especially when cell populations predicted to be vulnerable are targeted (e.g., OLs). While our results were similar between Pol III siRNA treatments, we did notice some deviation between conditions, especially at the level of OL-lineage markers and Pol III transcript expression data. While it is possible these differences reflect the known genotype–phenotype correlation seen in disease, it is unclear whether this is the true cause of variation seen here (Wolf et al., 2014; Gauquelin et al., 2019).

Our assessment of the precursor stage suggested migration was unaffected when *Polr3a, Polr3b,* or *Polr1c* were downregulated, proposing that failure of OPCs to migrate to myelination sites is unlikely to explain the hypomyelination seen in POLR3-related leukodystrophy. However, given that we did not completely downregulate Pol III subunit expression in our experiments, migration activity of OPCs may be affected in situations where Pol III expression is more heavily decreased.

To our surprise, targeting Pol III subunit expression in the precursor stage of development led to more EdU+ cells, suggestive of accelerated DNA synthesis. This is further supported by our Ki67 experiments, where Ki67 expression in cells at the same timepoint suggests a pure, proliferating OPC population. We hypothesize that a compensatory increase in OPC proliferation is seen in light of the differentiation and myelination defects, though further experiments are needed to confirm that this increased rate of DNA synthesis coincides with more cell division. Our results diverge from previous models implicating Pol III mutants of yeast, BHK-1 cells, and zebrafish gut development. While these models demonstrated a specific cell cycle stall at the G1-S phase transition leading to less proliferation, this may be due to OPC proliferation responding differently to hypomorphic Pol III compared to other cell types (Greco et al., 1989; Ittmann et al., 1993; Yee et al., 2007). Another factor to consider is that each of the cited models leverage different variants in genes encoding Pol III subunits, whereas our model specifically studies how Pol III subunit downregulation impacts OPC behaviour, which may be another reason our proliferation findings differ from prior observations.

OL-lineage cells with reduced expression of Pol III subunits exhibited impaired maturation when induced to differentiate, with reduced expression of late-stage OL and myelin transcripts and impaired branching complexity of OLs. Previous work suggested that defective OL development may be implicated in POLR3-related leukodystrophy pathophysiology (Choquet et al., 2019; Merheb et al., 2021). Our work puts forth a specific vulnerability of OL development to reduced Pol III activity in the immature OL stage. Transcriptomic data has previously shown Pol III subunit expression is dynamic during different stages of the OL-lineage (Zhang et al., 2014). Intriguingly, the expression of certain Pol III subunits (e.g., *Polr3b*, *Polr1c*) decreases in the post-mitotic phase, with OPCs having higher expression levels than newly formed OLs. These findings are perhaps counterintuitive as one would imagine that high protein synthesis demands during myelination (i.e., immature OL stage), both at the level of cytoskeletal proteins for process extension and myelin proteins for myelination, would require more Pol III activity (Zhang et al., 2014). However, this is not consistent, as *Polr3a* expression is slightly increased in newly formed OLs when compared to OPCs (Zhang et al., 2014). Expression of all three of these subunits (*Polr3a*, *Polr3b*, and *Polr1c*) decreases in mature OLs, although completion of myelination likely rationalizes why less Pol III would be required (Zhang et al., 2014). We hypothesize that immature OLs, predisposed with lower Pol III expression and high protein synthesis demands, are more vulnerable to hypomorphic Pol III, as seen in a disease context.

Consistent with our differentiation results, we noticed that reducing Pol III in OLs hindered the amount of myelin present, as inferred by fewer MBP+ regions in organotypic shiverer slice cultures and reduced MBP+ sheaths along synthetic fibers in co-culture with nanofibers. Our earlier findings would propose this hypomyelination is an outcome of a differentiation failure in the transition from precursor cells to OLs although it is possible that, in addition to a differentiation defect, an intrinsic deficit in myelin deposition could be contributing to this myelination finding.

To examine how Pol III transcriptional activity may relate to our cellular findings of impaired differentiation, we assessed Pol III transcript levels in siRNA-treated cells subjected to differentiation conditions. Pol III transcripts can be split into three categories based on their promoter type and basal transcription machinery used (Dieci et al., 2007). Type 1 transcripts include *5S* rRNA exclusively, while type 2 transcripts include tRNA genes, with some ncRNAs like *7SL* that use a hybrid type 2 promoter with upstream regulatory elements, also included in this category. Finally, type 3 promoters, which are unique in their use of an upstream promoter include ncRNAs such as *U6* (Dieci et al., 2007). We did not identify any changes in *5s, 7sl,* or *U6* expression suggesting these transcripts are not likely to be at play in our impaired differentiation findings. Previous work has revealed variable outcomes of a diseased state on these Pol III transcript levels. Indeed, *5S* expression has been shown to be unaffected in HEK293 cells harboring a pathogenic *POLR3A* variant associated with a typical POLR3-related leukodystrophy phenotype, increased in blood from patients with an atypical striatal form of POLR3-related leukodystrophy, and yet decreased in fibroblasts from patients with *POLR3K* mutations (Azmanov et al., 2016; Dorboz et al., 2018; Choquet et al., 2019). While the expression of *7SL* has often been shown to be decreased, as has been seen in blood samples from individuals with the striatal form of POLR3-related leukodystrophy and fibroblasts from individuals with biallelic pathogenic *POLR3K* variants, this finding is not consistent, as in fibroblasts from patients with a typical disease course, *7SL* was unaffected (Shimojima et al., 2014; Azmanov et al., 2016; Dorboz et al., 2018; Choquet et al., 2019). Type 3 transcripts have often been shown to be unaffected in a disease state. In fact, previous work has revealed the upstream promoter utilized by type 3 transcripts can be occupied, and may therefore receive compensatory transcription by RNA Polymerase II, supporting a rationale for why some Pol III transcripts may be unaffected by reduced expression of a Pol III subunit (James Faresse et al., 2012). Further, the focus on samples from individuals with different disease severities, use of various patient sample types (e.g., fibroblasts, blood) and different analysis techniques, likely rationalize this variability in Pol III expression levels.

There are two main hypothesized disease mechanisms pertaining to how Pol III transcription might relate to POLR3-related leukodystrophy, which may not be mutually exclusive. The first, that certain ncRNAs may be required for OL-lineage development and myelination, and the second, that tRNA homeostasis might be altered in a diseased state, leading to insufficient translation and therefore protein synthesis during critical timepoints in development (Thiffault et al., 2015; Choquet et al., 2019; Lata et al., 2021). One such ncRNA that has been suggested as playing a role in the disease state is *BC200* RNA, a brain-enriched transcript thought to play a role in neurons by local translation of mRNA in dendrites (Dieci et al., 2007; Choquet et al., 2019). In MO3.13 BC200^KO^ cells, there is reduced expression of *MBP* mRNA upon differentiation (Choquet et al., 2019). However, glial transcriptomic profiling has revealed that *BC200* is not normally expressed in human OLs, and neither is the functional analog *Bc1* RNA in mouse OLs, suggesting the *BC200* expression in MO3.13 cells could be due to the cancerous origin of these cells (McLaurin et al., 1995; Zhang et al., 2014; Booy et al., 2017). While this does not preclude that another, unidentified, Pol III transcript might be relevant for OL maturation and/or myelination, or that BC200 may contribute to neuronal phenotypes (e.g., cerebellar or striatal involvement) seen in a diseased state, it is improbable to explain the cellular pathology seen here.

Various studies on the transcriptional impact of POLR3-related leukodystrophy both in patient samples and model organisms have supported involvement of tRNAs in the pathophysiology of the disease (Arimbasseri et al., 2015; Azmanov et al., 2016; Dorboz et al., 2018; Choquet et al., 2019). Our findings further support this; we found that Pol III siRNA-treated OL-lineage cells induced to differentiate trended towards a decrease in the levels of distinct tRNAs, which was significant in the siPolr3a condition. The identification of hypomyelinating leukodystrophies that arise from biallelic variants in genes encoding cytoplasmic aminoacyl tRNA synthetases strengthens a tRNA involvement in disease, as does the broader connection between tRNAs and neurological disorders (Taft et al., 2013; Borck et al., 2015; Nafisinia et al., 2017; Mendes et al., 2018; Schaffer et al., 2019). Importantly, changes in the tRNA pool could have tissue and even lineage-specific effects based on codon usage bias in these cell types, and further transcript-specific effects based on codon preference in mRNAs, suggesting a possible mechanism for our findings of a defect in the transition of OL-lineage stages (Hanson and Coller, 2018; Watt et al., 2022).

Moreover, tRNA availability for protein arginylation is an additional way tRNAs may be important for OL maturation and myelination. The transfer of arginine from tRNA onto proteins, or arginylation, is a post-translational modification catalyzed by arginyl-protein-transferase 1 (Ate1) and has recently been shown to play a critical role in OL maturation (Palandri et al., 2022). Various cytoskeletal regulators of OL maturation are subject to arginylation and in primary cultures of mouse OLs with a genetic ablation in Ate1, branching complexity was significantly reduced while *in vivo,* these mice showed fewer mature (MOG+ or MBP+) OLs, which the authors suggested could be due to impaired OL differentiation (Palandri et al., 2022). It is interesting to note that biallelic variants of the cytoplasmic arginine aminoacyl-tRNA synthetase (*RARS1*) are associated with a hypomyelinating leukodystrophy (Nafisinia et al., 2017).

Oligodendrocytes bear large transcriptional and translational demands which could put them at an increased vulnerability in the face of altered tRNA homeostasis and a potential protein synthesis defect (Jahn et al., 2020). Second to canonical myelin proteins, cytoskeletal proteins constitute a major demand for OLs and are synthesized earlier in the lineage (Lee and Hur, 2020). Notably, biallelic variants in genes encoding OL cytoskeletal proteins are implicated in hypomyelination, with variants in *TUBB4A* (encoding tubulin ß4, a microtubule specific to OLs) predicted to alter tubulin polymerization and microtubule stability, causing hypomyelination with atrophy of the basal ganglia and cerebellum, a distinct hypomyelinating leukodystrophy (Simons et al., 2013). The OL cytoskeleton is not only critical for OL maturation but for myelination as microtubules mediate the transport of *Mbp* mRNA to process terminals (Ainger et al., 1993). Cytoskeletal proteins are also regulated, including by the myelin protein CNPase which has been shown to drive microtubule assembly, enabling OL process outgrowth, with OLs derived from CNP-deficient mice demonstrating smaller and fewer processes *in vitro* (Lee et al., 2005). It is possible that in the face of reduced Pol III expression, immature OLs are unable to meet the high protein synthesis demands of cytoskeletal and/or myelin proteins, impacting the development and function of these cells.

Finally, the data herein suggest the Pol III siRNA-treated OLs experiencing impaired differentiation are not subjected to apoptosis, a process known to regulate the maturation of OL-lineage cells. This finding is consistent with previous work, which found that caspase 3 was not activated in brain lysates of a *Polr3a* mutant mouse (Merheb et al., 2021). Crucially, this would suggest that OLs exhibiting defective differentiation in the context of decreased Pol III could be rescued by putative therapeutics.

In summary, our study identifies a role for Pol III in OL development, with decreased Pol III expression leading to defects at the level of OL-lineage maturation, including an increased proportion of proliferating OPCs, impaired differentiation into mature OLs and concomitantly, hindered myelination. We likewise pinpoint alterations at the level of Pol III transcriptional activity which coincide with our cellular findings and therefore provide insight as to what is occurring molecularly to help explain the cellular defects seen. Nevertheless, a more detailed analysis of Pol III transcriptional activity in POLR3-HLD models is warranted and will hopefully deepen our molecular understanding of this disease. Notably, as we were able to identify cellular and transcriptional deficits with a 60% reduction in RNA, our results suggest that Pol III subunit expression greater than 40% is compulsory for normal OL development. In turn, our results shed light on pathophysiological mechanisms underlying hypomyelination in POLR3-related leukodystrophy, providing foundational knowledge relevant to the field’s advancement in developing therapeutics.

## Data availability statement

The original contributions presented in the study are included in the article/Supplementary material, further inquiries can be directed to the corresponding author.

## Ethics statement

The animal study was reviewed and approved by Animal Resource Division of the Research Institute of the McGill University Health Centre (protocol #2018-8055).

## Author contributions

JM designed and conceptualized the study, contributed to the acquisition of data, performed analysis and interpretation of data, and drafted and revised the manuscript for intellectual content. MMR designed and conceptualized the study, contributed to the acquisition of data, performed analysis and interpretation of data, and revised the manuscript for intellectual content. XC contributed to the acquisition of data, performed analysis and interpretation of data, and revised the manuscript for intellectual content. GB designed and conceptualized the study, contributed to the acquisition of data, performed analysis and interpretation of data, drafted and revised the manuscript for intellectual content, and supervised the study. All authors contributed to the article and approved the submitted version.

## Funding

This research was funded by research grants from the Montreal Children’s Hospital Foundation, Leuco Action, and Fondation les Amis d’Elliot. This research was undertaken thanks in part to funding from the Canada First Research Excellence Fund and Fonds de recherche du Québec, awarded to the Healthy Brains, Healthy Lives (HBHL) initiative at McGill University. JM is supported by HBHL. MMR would like to acknowledge Vanier Canada Graduate Scholarships and the McGill Faculty of Medicine and Health Sciences for financial support. GB has received a Clinical Research Scholar Junior 1 award from the Fonds de Recherche du Quebec – Santé (FRQS) (2012–2016), the New Investigator Salary Award from the Canadian Institutes of Health Research (2017–2022), and the Senior Clinical Research Scholar award from the FRQS (2022–2025).

## Conflict of interest

GB is/was a consultant for Passage Bio Inc. (2020–2022) and Ionis (2019). She is/was a site investigator for the Alexander’s disease trial of Ionis (2021-present), Metachromatic leukodystrophy of Shire/Takeda (2020–2021), Krabbe and GM1 gene therapy trials of Passage Bio (2021-present), Passage Bio GM1 natural history study (2021-present) and Adrenoleukodystrophy/Hematopoietic stem cell transplantation natural history study of Bluebird Bio (2019), a site sub-investigator for the MPS II gene therapy trial of Regenxbio (2021-present) and the MPS II clinical trial of Denali (2022-present). She has received an unrestricted educational grant from Takeda (2021–2022). She serves on the scientific advisory board of the Pelizaeus-Merzbacher Foundation, the Yaya Foundation Scientific and Clinical Advisory Council and is the Chair of the Medical and Scientific Advisory Board of the United Leukodystrophy Foundation. She is a member of the Vanishing White Matter Consortium, the H-ABC Clinical Advisory Board and the Chair of the POLR3-related (4H) Leukodystrophy Consortium. She is on the editorial boards of Neurology Genetics, Frontiers in Neurology – Neurogenetics, and Journal of Medical Genetics.

The remaining authors declare that the research was conducted in the absence of any commercial or financial relationships that could be construed as a potential conflict of interest.

## Publisher’s note

All claims expressed in this article are solely those of the authors and do not necessarily represent those of their affiliated organizations, or those of the publisher, the editors and the reviewers. Any product that may be evaluated in this article, or claim that may be made by its manufacturer, is not guaranteed or endorsed by the publisher.
