# Supplementary material for: Decreased RNA polymerase III subunit expression leads to defects in oligodendrocyte development
Source: Front Neurosci. 2023 Apr 25;17:1167047. doi: 10.3389/fnins.2023.1167047 (PMC10167296; doi:10.3389/fnins.2023.1167047)
Supplement: Supplementary file 1 [file Data_Sheet_1.docx]

**Decreased RNA polymerase III subunit expression leads to defects in oligodendrocyte development**

**Supplemental Figures and Tables**

Julia Macintosh MSc^1,2^, Mackenzie Michell-Robinson MSc^1,2^, Xiaoru Chen PhD^1,2^, Geneviève Bernard MD, MSc, FRCPc^1,2,3,4,5*^

**Affiliations**

^1^Department of Neurology and Neurosurgery, McGill University, Montreal, Quebec, Canada

^2^Child Health and Human Development Program, Research Institute of the McGill University Health Center, Montreal, Quebec, Canada

^3^Department of Pediatrics, McGill University, Montreal, Quebec, Canada

^4^Department of Human Genetics, McGill University, Montreal, Quebec, Canada

^5^Department Specialized Medicine, Division of Medical Genetics, McGill University Health Center, Montreal, Quebec, Canada

**Correspondence**: Geneviève Bernard, MD, MSc, FRCPc, Research Institute of the McGill University Health Centre, 1001 boul. Décarie, Site Glen Pavilion E/Block E, Montreal, QC H4A 3J1, Canada; [genevieve.bernard@mcgill.ca](mailto:genevieve.bernard@mcgill.ca)

**Figure S1:** Representative immunofluorescence images (from three independent experiments with similar results) of siRNA-treated OPCs in proliferation media, showing PDGFRα (green), NG2 (red), and Ki67 (yellow). Nuclei stained with DAPI. Separate fluorescent channels shown. Scale bar, 50 µm (20X).

**
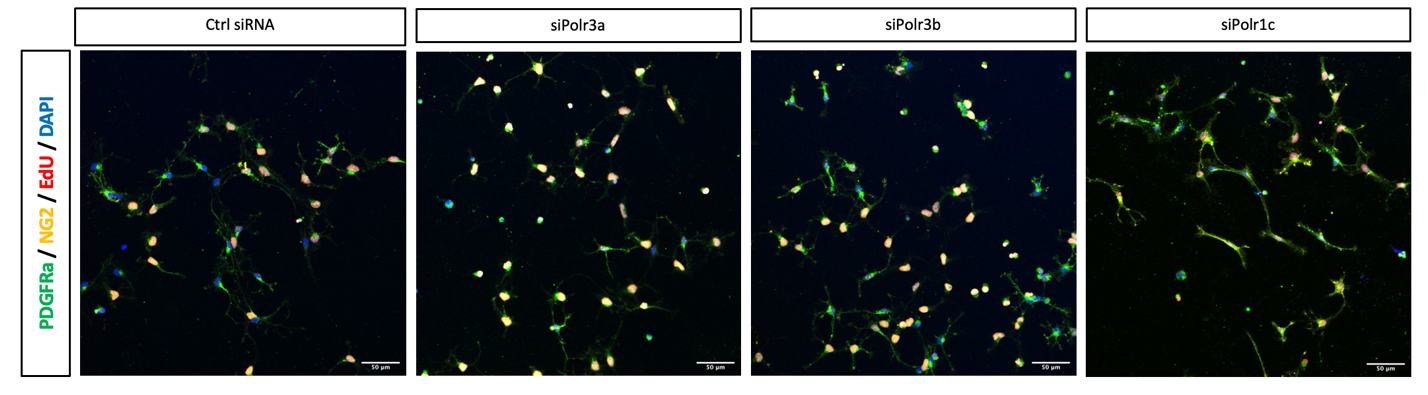
**

**Figure S2:** Representative images (from five independent experiments with similar results) of siRNA-treated OPCs in proliferation media labeled with EdU and showing PDGFRα (green) and NG2 (yellow). Nuclei stained with DAPI. Scale bar, 50 µm.

**Figure S3:** Representative images (from five independent experiments with similar results) of siRNA-treated OPCs labelled with EdU. Nuclei stained with DAPI. Separate fluorescent channels shown. Scale bar, 50 µm (20X).

**Table S1:** Antibody information

| Anti-Ki67 (1:200) | Novus | AF7649 |
| --- | --- | --- |
| Anti-PDGFRα (ICC 1:100, IP 1:250) | BD | 558774 |
| Anti-NG2 (1:200) | Millipore | AB5320 |
| Anti-MOG (1:300) | R&D | MAB2439 |
| Anti-MBP (1:100-1:500) | Novus | NB600-717 |
| Anti-Calbindin D28-k (1:5000) | Swant | CB38 |
| Anti-Cleaved caspase 3 (1:250) | CST | 9661 |
| Anti-O4 (1:200) | R&D | MAB1326 |
| AlexaFluor 488 donkey anti-rat (1:1000) | Fisher Scientific | A21208 |
| AlexaFluor 647 donkey anti-rabbit (1:300-1:500) | Jackson ImmunoResearch | 711-605-152 |
| AlexaFluor 594 donkey anti-sheep (1:500) | Abcam | 713-585-147 |
| AlexaFluor 555 donkey anti-goat (1:500) | Invitrogen | A-21432 |
| Goat anti-rat IgG (IP 1: 333) | Jackson ImmunoResearch | 115-005-167 |
| AlexaFluor 594 donkey anti-mouse (1:500) | Jackson Immunoresearch | 715-585-020 |

**Table S2:** RT-qPCR primers

| *Polr3a* | F: CATGTCGGGTACTTCAGGGCT |
| --- | --- |
|  | R: TAATGTGGCAGCAGGTTTTGCAG |
| *Polr3b* | F: TTGGAGCCTCAGTTACCAGC |
|  | R: GAATCGACCCTGTTTCACGG |
| *Polr1c* | F: GTCCACACCACGGACTTTC |
|  | R: CCACACGGAAATTCTTCTCGA |
| *Pdgfr*α | F: CATCTATGTACCAGACCCAGAC |
|  | R: AAGGTATGATGGCAGAGTCATCC |
| *Cspg4* | F: CCTTCCTACAAGTGACCATTGCC |
|  | R: TCCTGGACCACCTCTAGCT |
| *Fyn* | F: GAAGCCCGCTCCTTGACA |
|  | R: CCAAGTTTTCCAAAGTACCACTCTT |
| *Nfasc* | F: AATCCGAGTCCTGAACAGCA |
|  | R: TTCCCTCCAGTAGTAAGCTCGATA |
| *Kif19a* | F: GCTGAGTGACAAGGGCAGTAA |
|  | R: GTTCCCGCCTAGTGAATCCTTC |
| *Mbp* | F: TCAAGAACATTGTGACACCTCG |
|  | R: AGCTAAATCTGCTGAGGGAC |
| *Mog* | F: GCCTGTTTGTGGAGCTTCTCTTG |
|  | R: CCTATCACTCTGAATTGTCCTGCAT |
| *Cnp* | F: CTGAGACCCTCCGAAAAGCTG |
|  | R: TCCAGAAATAAAGTGTCGAAGCTCT |
| *5s* | F: GCCATACCACCCTGAACGC |
|  | R: TATTCCCAGGCGGTCTCCC |
| *7sl* | F: GGAGTTCTGGGCTGTAGTGC |
|  | R: TTTGACCTGCTCCGTTTCCG |
| *U6* | F: CGCTTCGGCAGCACATATAC |
|  | R: TTCACGAATTTGCGTGTCAT |
| tRNA-iMet-CAT-1-2 | F: GCAGCGGAAGCGTGCT |
|  | R: AGCAGAGGATGGTTTCGATCC |
| tRNA-Tyr-GTA-1-1 | F: CTCAGTTGGTAGAGCGGAGG |
|  | R: CCTTCGAGCCGGATTCG |
| tRNA-Lys-TTT-2-1 | F: CCTGGATAGCTCAGTCGGTAGAG |
|  | R: GACTTGAACCCTGGACCCTCA |
| *18s* | F: GTAACCCGTTGAACCCCATT |
|  | R: CCATCCAATCGGTAGTAGCG |
| *Gusb* | F: GTGGTATGAACGGGAAGCAAT |
|  | R: AACTGCATAATAATGGGCACTGT |
| *Pgk1* | F: TGGTGGGTGTGAATCTGCC |
|  | R: ACTTTAGCGCCTCCCAAGATA |

**Table S3:** Materials and Reagents

| **Reagent** | **Source** | **Identifier** |
| --- | --- | --- |
| Griffonia (Bandeiraea) simplicifolia lectin I GSL I, BSL I | Biolynx | VECTL1100 |
| Papain | Worthington | LS003126 |
| DNase I | Worthington | LS002007 |
| L-cysteine | Sigma | C7477 |
| Insulin from bovine pancreas | Sigma | I6634 |
| Ovomucoid | Worthington | LS003086 |
| Bovine Serum Albumin | Sigma | A3912 |
| Transferrin | Sigma | T1147 |
| Putrescine | Sigma | 221732 |
| Progesterone | Sigma | P8783 |
| Sodium selenite | Sigma | S5261 |
| Penicillin-Streptomycin | Wisent | 450-201-EL |
| Sodium pyruvate | Gibco | 11360070 |
| Glutamax | Gibco | 35050061 |
| N-acetyl-L-cysteine | Sigma | A7250 |
| Forskolin | Sigma | F3917 |
| D-Biotin | Sigma | B4639 |
| Trace Elements B | Fisher Scientific | MT99175Cl |
| B-27 without vitamin A | ThermoFisher | 12587010 |
| Recombinant human PDGF-AA | Preprotech | 100-13A |
| Recombinant human bFGF | Preprotech | 100-18B |
| Triiodothyronine | Sigma | T6397-100mg |
| D-PBS with Mg^2+^ Ca^2+^ | ThermoFisher | 14287080 |
| Earle’s Balanced Salt Solution | Wisent | 311210CL |
| Dubleco’s Modified Eagle’s Medium | Wisent | 319-005-CL |
| Minimum Essential Medium | Sigma | 51412C-1000ML |
| DMEM-F12 1% GlutaMAX | ThermoFisher | 10565018 |
| Fetal Bovine Serum | Wisent | 089-150 |
| Heat-Inactivated horse serum | ThermoFisher | 26050088 |
| Glucose | ThermoFisher | A2494001 |
| Fungizone | ThermoFisher | 15290018 |
| B-27 Supplement (50X), serum free | ThermoFisher | 17504044 |
| N-2 Supplement (100X) | ThermoFisher | 17502048 |
| Poly-d-Lysine hydrobromide | Sigma | P8099 |
| Trypsin/EDTA | Wisent | 325.043-cl |
| Corning™ Transwell™ Multiple Well Plate with Permeable Polycarbonate Membrane Inserts | Fisher | 07-200-150 |
| ReadyProbes™ Cell Viability Imaging Kit, Blue/Green | Invitrogen | R37609 |
| TRIzol^TM^ reagent | ThermoFisher | 15596018 |
| miRNeasy Mini Kit | Qiagen | 217004 |
| RNAse free DNAse kit | Qiagen | 79254 |
| Oligo(d)T | ThermoFisher | 18418020 |
| Random Hexamers | IDT | 51-01-18-25 |
| RNasin® Ribonuclease Inhibitors | Promega | N2611 |
| MMLV RT | Promega | M1705 |
| MMLV 5x reaction buffer | Promega | M5313 |
| SsoAdvanced Universal SYBR® Green Supermix | Bio-Rad Laboratories | 1725274 |
| GoTaq® Master Mixes | Promega | M7122 |
| Extracta™ DNA Prep for PCR | QuantaBio | CA97065-350 |
| MilliporeSigma™ Millicell™ Culture Plate Inserts | Fisher | PICM03050 |
| Pre-Treated German Glass Coverslips 15mm #1 | Electron Microscopy Sciences | 72291-04 |
| Thermo Scientific™ Shandon™ Immu-Mount™ | Fisher Scientific | 9990402 |
| Click-iT™ EdU Cell Proliferation Kit for Imaging | ThermoFisher | C10340 |
| siGENOME mouse Polr3a siRNA - SMARTPool 5nmol | Dharmacon | M-160178-00-0005 |
| siGENOME mouse Polr3b siRNA - SMARTPool 5nmol | Dharmacon | M-064863-00-0005 |
| siGENOME mouse Polr1c siRNA - SMART Pool, 5nmol | Dharmacon | M-046056-01-0005 |
| siGENOME Non-Targeting siRNA Control Pools | Dharmcon | D-001206-14-05 |
| Lipofectamine | Invitrogen | 13778150 |
| 96-well plates with Mimitex aligned fibers (PLLA, 2µm fiber diameter, 2-4µm thick) | AMSBIO | AMS-TECL-005-8X |
